# Construction Notes

**Published:** 1893-01-14

**Authors:** 


### CONSTRUCTION NOTES.

A NEW pavilion, with two wards containing fifty-two beds, is to be added to the Royal Hospital for Incurables, Dublin.

THE directors of the Dundee Royal Infirmary propose to erect a new operating theatre and other offices. The cost of these alterations is estimated at £119 12s. 6d.

SEVERAL alterations and improvements have been recently made in the heating, cooking, and washing departments of the Convalescent Home in connexion with the Dundee Royal Infirmary. The expense of these alterations has been borne by Mrs. Gilroy.

IT is proposed to erect a temporary hospital for infectious diseases at Woodhouse, Keighley, at an estimated cost of £650. The building is to be of corrugated iron, lined with boards, with an accommodation for twelve beds. Messrs. Judson and Moore are the architects.

AT the annual meeting of the Guest Hospital, Dudley, on the motion of Mr. E. Smith, seconded by Dr. Massiter, a resolution was carried to the effect that steps be taken at once to provide a private ward and operating-room for the female sick of the building, and the meeting pledged itself to raise £400, or the sum needed in addition to £400 already in hand.

THE committee of the Royal Albert Hospital, Devonport, have decided to build a new operating theatre, with easy access by means of a lift to the wards of the hospital. A contract has been taken for £1,209 15s. for the new building and alterations, and the hydraulic lift will cost another £400. While this work is being done the committee propose taking advantage of the opportunity to improve still further the sanitary arrangements of the hospital.

